# Microbiome-Based Therapeutics for Salt-Sensitive Hypertension: A Scoping Review

**DOI:** 10.3390/nu17050825

**Published:** 2025-02-27

**Authors:** Abdulwhab Shremo Msdi, Anahita Haghparast, Kevin W. Garey, Elisabeth M. Wang

**Affiliations:** Department of Pharmacy Practice and Translational Research, College of Pharmacy, University of Houston, 4349 Martin Luther King Boulevard, Houston, TX 77204, USA

**Keywords:** microbiota, live biotherapeutic products, probiotics, prebiotics, postbiotics, cardiovascular disease

## Abstract

The purpose of this scoping review was to provide a comprehensive understanding of the current knowledge concerning the gut microbiome and SCFAs as emerging treatments for salt-sensitive hypertension. Relevant animal and human studies were identified via PubMed through August 2024. Twenty-four human (*n* = 9) and animal (*n* = 15) trials were included. Most human studies were observational (*n* = 6), aiming to compare gut microbiota differences between hypertensive and normotensive individuals. Three human studies evaluated microbiome-based interventions either via a sodium-restricted diet (*n* = 2) or prebiotic supplementation (*n* = 1). Fifteen animal trials involving either mice or rats were identified, all of which were interventional. These included dietary changes (*n* = 9), probiotic treatments (*n* = 1), postbiotic primarily bacterial metabolites (*n* = 4), and live biotherapeutic products (*n* = 4). All interventions were effective in decreasing blood pressure. Microbiome-based therapies as biologic modifiers for salt-sensitive hypertension are emerging. Substantial knowledge gaps remain, warranting further research to fully explore this promising therapeutic avenue.

## 1. Background

Hypertension (HTN) is a significant public health concern, affecting nearly half of USA adults and contributing to over 124,000 deaths annually, highlighting its substantial impact on the nation’s disease burden [1]. HTN is a complex disease influenced by both genetic and environmental factors. Among the environmental factors, high dietary salt (HDS) intake is recognized as a major contributor to elevated blood pressure (BP) [2]. Approximately 50–75% of individuals with HTN exhibit salt sensitivity, characterized by a rise in BP of 10 mm Hg or more in response to excessive dietary salt intake [3,4,5]. This makes it one of the most common clinical HTN phenotypes. Additionally, it is emphasized that there is a subgroup of patients who are particularly responsive to dietary and microbiome-targeted interventions [6,7]. Initially, two primary mechanisms were proposed to explain salt-sensitive HTN: renal dysfunction and vascular dysregulation [3]. However, gut dysbiosis, defined as an imbalance in the gut microbiota or its metabolites, may also affect salt-sensitive HTN [8]. For example, germ-free mice given fecal microbiota transplantation (FMT) from hypertensive patients exhibited gut dysbiosis and elevated BP [9]. The gut microbiome is a complex ecosystem of microorganisms that contributes to overall health, including the regulation of BP [4,8]. One key function of the gut microbiome is the production of short-chain fatty acids (SCFAs), metabolic byproducts that are essential for human health [4,8]. The human body has approximately 17 enzymes dedicated to digesting dietary fibers into SCFAs, whereas the gut microbiome harbors thousands of enzymes capable of fermenting these complex resistant starches [10]. From this fiber fermentation process, SCFAs such as acetate, butyrate, and propionate are produced in the gut lumen [11,12]. SCFAs can be systemically absorbed and exert effects through G-protein-coupled receptors (GPCRs), notably Gpr41/FFAR3 and Olfr78/OR51E2, which are abundantly expressed in vascular endothelial cells and the juxtaglomerular complex and play a critical role in regulating BP [13].

Disruptions in the balance of the gut microbiome and SCFA production can result in HTN development by altering these BP regulation mechanisms [14]. Understanding the biological effect of the gut microbiome and SCFAs’ interplay on salt-sensitive HTN can offer insights into the mechanisms underlying salt-sensitive HTN and endothelial dysfunction. This scoping review aimed to explore the existing research on salt-sensitive HTN and the gut microbiome with a particular focus on emerging therapies. By synthesizing the current knowledge, this review will inform future studies and explore the potential of targeting the gut microbiome composition and its metabolites as a novel therapeutic approach to managing HTN.

## 2. Methods

This scoping review was conducted following the JBI methodology to map and synthesize current research on the relationship between salt-sensitive HTN, the gut microbiome, and SCFAs [15]. The goal of this scoping review was to identify existing research exploring the impact of the gut microbiome or SCFAs on the pathophysiology of salt-sensitive HTN. We developed our search strategy to answer the following questions: (i) Which animal or human experimental models have investigated salt-sensitive HTN and the gut microbiome or SCFAs? (ii) Which interventions have been assessed?

A search was conducted on PubMed for articles through August 2024 using the keywords “microbiome” and “hypertension”. All study designs (animal and human) investigating the relationship between the gut microbiome and salt-sensitive HTN in animal and human models were included. Studies evaluating alternative etiologies, review articles, and non-English studies were excluded. The bibliographies of selected articles were screened for relevant primary studies. Prior to screening, study duplicates were manually removed. Two reviewers (AH and ASM) independently screened the titles and abstracts of the collected articles to identify those meeting the inclusion criteria. The full texts of articles meeting the inclusion criteria were screened by the same investigators (AH and ASM). Discrepancies between the two reviewers were resolved by a third investigator (KWG or EMW). For human trials, the extracted data included the study design types, population studied, type of intervention, effect on BP, microbiome, and SCFA results. For animal studies, the data extracted included the population studied, type of intervention, effect on BP, microbiome, and SCFA results. Treatments were categorized as dietary interventions, prebiotics (non-digestible food ingredient that stimulates the growth of colonic bacteria), probiotics (microorganisms that confer a health benefit when given in the appropriate amount), postbiotics (bioactive compounds produced by gut microorganisms), or live biotherapeutic products (biologic medicinal products with live microorganisms as active substances) [16,17,18].

## 3. Results

**Figure 1:**
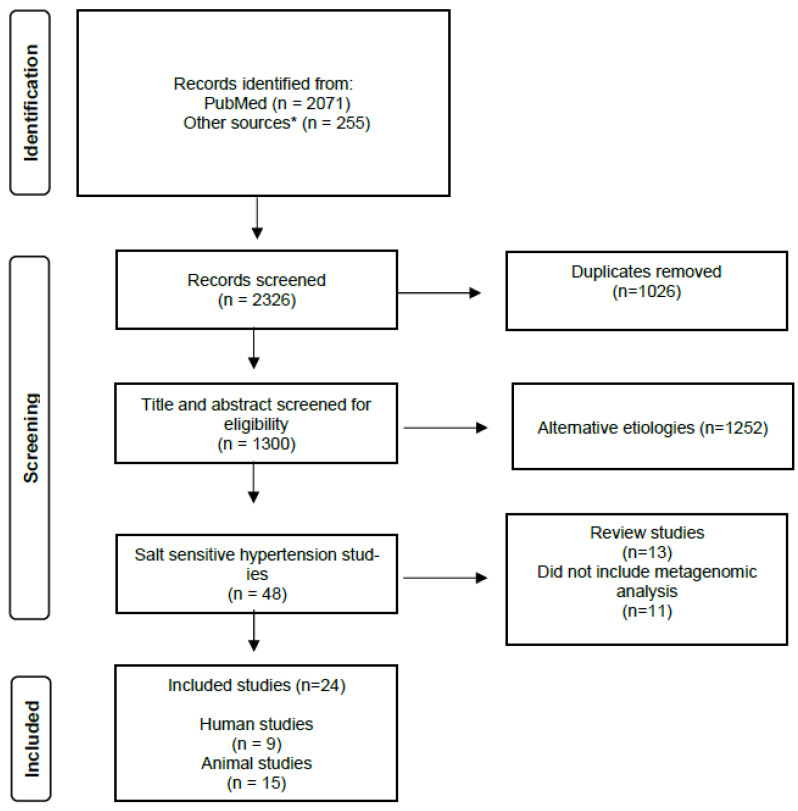
Study selection flow diagram. * Review studies’ bibliographies were screened for relevant primary studies.

The search yielded 2071 studies, with an additional 255 studies found through backward reference screening. After removing duplicates, 1300 articles remained for title and abstract screening. Among these, 48 articles examined the relationship between the gut microbiome or SCFA and salt-sensitive HTN. Following a full-text review, 24 studies met the eligibility criteria, including 9 human trials and 15 animal studies. A detailed summary of the search results and study selection process is presented in Figure 1.

### 3.1. Human Studies

Nine human trials, all involving adult populations, were identified (Table 1). Most studies (*n* = 6) were observational, with the primary objective of demonstrating microbiota differences between hypertensive patients and healthy controls. Hypertensive patients consistently exhibited reduced bacterial diversity, while normotensive individuals were enriched with fiber-metabolizing bacteria associated with butyrate production [19,20]. One study identified an epidemiologic link between high salt intake and increased BP that was correlated with the bacterial genus *Prevotella*, being more prevalent in hypertensive patients [21]. Three studies evaluated microbiome-targeted interventions, including a sodium-restricted diet (*n* = 2) and prebiotic supplementation (*n* = 1) [6,7,20]. A phase I trial in healthy male volunteers revealed that a 2-week HSD elevated BP and decreased gut *Lactobacillus* species survival [6]. A sodium-restricted diet in untreated hypertensive patients reduced BP, especially in females, and increased the systemic SCFA concentrations [7]. Finally, a randomized crossover trial demonstrated that supplementation with acetylated and butyrylated high-amylose maize starch (HAMSAB) lowered BP, elevated the systemic acetate and butyrate levels, and increased the abundance of *Bacteroides* and *Ruminococcus* [20]. These findings collectively highlight the potential of dietary interventions, such as sodium restriction and prebiotic supplementation, to modulate the gut microbiome and improve BP outcomes.

### 3.2. Animal Studies

Fifteen animal trials involving either mice or rats were identified (Table 2). Specific pathogen-free conditions were maintained throughout all animal experiments. All studies were interventional and included one or more interventions, including dietary interventions (*n* = 9), probiotic treatments (*n* = 1), postbiotic bacterial metabolite administration (*n* = 4), and live biotherapeutic products (*n* = 4). Dietary interventions primarily involved salt restriction (*n* = 3) or dietary fiber supplementation (*n* = 4). All interventions were effective in decreasing BP. Notably, FMT was utilized by all live biotherapeutic product trials. FMT from salt-sensitive hypertensive donors consistently increased BP in recipient controls, except in one study by Mell et al., where FMT from salt-resistant rats further exacerbated HTN and elevated plasma acetate in salt-sensitive rats on a HSD [24]. Another study found increased fecal acetate and propionate levels in rats fed an HSD [25]. Microbiome changes were examined in 14 studies, highlighting potential protective roles for *Bacteroides*, *Parabacteroides*, *Lactobacillus*, *Bifidobacterium*, and *Akkermansia* in 8 of 14 (57%) trials. These findings underscore the potential of microbiome-targeted interventions, including dietary modifications and FMT, in mitigating salt-sensitive HTN and promoting gut microbial resilience.

## 4. Discussion

This review offers insights into the interaction between diet, the gut microbiome, SCFA levels, and changes in BP. Several microbiome-based therapeutics were identified, with only a few having progressed to human trials. A HSD was consistently associated with gut dysbiosis and the subsequent exacerbation of salt-sensitive HTN, while a high-fiber diet and elevated the SCFA plasma levels attenuated the elevated BP response. Salt-induced gut dysbiosis was characterized by the depletion of beneficial bacteria (*Lactobacillus*, *Bifidobacterium*, and *Bacteroidetes*) and increased *Prevotella.* This is thought to activate proinflammatory pathways and contribute to persistent HTN [6,21,31,35]. Notably, *Lactobacillus* and *Bifidobacterium* are involved in the metabolism of fecal tryptophan into indole derivatives, which have been shown to reduce inflammation and BP [6,31]. Furthermore, a lack of butyrate-producing bacteria has been associated with colonic inflammation, an impaired gut epithelium, and elevated SBP, which can be normalized by butyrate or fiber supplementation [19,20].

**Figure 2:**
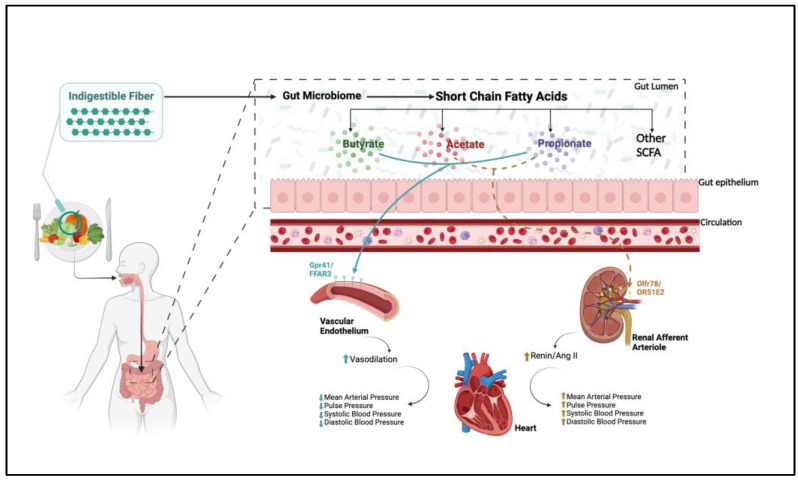
Illustration of the conversion of indigestible fiber into short-chain fatty acids and subsequent systemic impact via G-protein-coupled receptors. Straight arrow (Blue) indicates direct activation. Dashed arrow (Brown) indicates negative feedback loop.

Another mechanism involves HSD-induced endothelial dysfunction [7,34]. Liu et al. demonstrated that a HSD significantly elevated the plasma endothelin-1 levels and decreased nitric oxide in wild-type mice, compared to those on a standard-salt diet, leading to a marked increase in BP [34]. Dietary fibers, such as resistant starch, function as prebiotics and undergo fermentation by commensal bacteria in the large intestine. This fermentation process generates SCFAs, which have been shown to possess antihypertensive properties [13,20]. A high-fiber diet increases the abundance of SCFA-producing bacteria and elevates the SCFA plasma levels, potentially mitigating salt-sensitive HTN [12,20,28,29]. Acetate, butyrate, and propionate are the most abundant and well-characterized SCFAs that act systemically on GPCRs, such as Gpr41 and Olfr78 [13]. Gpr41, located in the vascular endothelium, exerts a direct vasodilatory effect, and Olfr78, expressed in the juxtaglomerular cells of the afferent arteriole, stimulates renin release, leading to increased BP [36,37]. Although these two receptors exert contrasting physiological responses to the same stimuli, the varying binding affinities between SCFAs and these receptors create a negative feedback loop, maintaining homeostasis and preventing significant fluctuations in BP (Figure 2) [38]. Both of these mechanisms appear to be amenable to therapeutic interventions.

This review identified key gaps in the literature regarding the relationship between dysbiosis and salt-sensitive HTN. Most of the available data are derived from animal models, and human studies are urgently needed to move the field forward. Most human studies were observational, non-interventional, or limited by small sample sizes. In contrast, numerous animal studies have demonstrated promising microbiome-targeted interventions, such as salt-restricted diets and fiber supplementation, which are considered safe and could rapidly transition to clinical trials. Larger, interventional studies specifically designed to address targeted research questions are necessary.

Human and animal studies indicate that SCFAs play a key role in linking gut dysbiosis to HTN [7,12,20,29]. Dietary fibers are a key modulator, enabling the targeted manipulation of the gut microbial composition to enhance SCFA production profiles, potentially addressing different HTN etiologies [39]. However, a critical gap remains in understanding how specific bacterial taxa influence SCFA production and BP regulation. Identifying SCFA-producing bacteria and their response to diet could lead to further microbiota-based interventions for HTN. This has been shown in an animal model, but further data in humans are required. Data directly assessing the impact of antibiotics on the gut microbiome and BP control in salt-sensitive HTN are lacking. Interestingly, a prospective, pilot, open-label study demonstrated that minocycline reduced BP in high-risk patients with resistant HTN and decreased neuroinflammation [40]. Yang et al. reported that a 4-week minocycline regimen restored BP control (*p* < 0.01) and decreased the F/B ratio (*p* < 0.01) in angiotensin II-infused rats [22]. The validation of these findings in patients with HTN is needed. Finally, while this review suggests that gut dysbiosis can affect BP regulation, data from hypertensive rat models challenge the direction of this relationship. Reciprocal FMT experiments between spontaneously hypertensive rats (SHR) and wild-type rats imply that the host’s uncontrolled BP might influence the gut microbiome more than the microbiome affects BP [41]. Nevertheless, these studies did not find significant changes in gut function, as measured by SCFAs, between the two groups. This complicates our understanding of the microbiome’s role in these experiments.

In summary, this review highlights the link between diet, gut dysbiosis, and salt-sensitive HTN. Dietary fibers and SCFAs may have protective effects. Most evidence comes from animal studies, and limited human research supports these findings, highlighting the need for larger, well-designed human trials. Furthermore, a better understanding of how antibiotics affect gut imbalances and BP regulation in salt-sensitive HTN is needed.

Figure 2 provides an illustration of the conversion of indigestible fiber into short-chain fatty acids and the subsequent systemic impact via G-protein-coupled receptors. Indigestible fibers are fermented by the gut microbiome and are metabolized into short-chain fatty acids (SCFAs) such as acetate, butyrate, and propionate. These primary SCFAs then traverse the gut epithelium and enter the circulation, interacting with G-protein-coupled receptors GPR41/FFAR3 in the vascular endothelial cells, promoting vasodilation and reducing the blood pressure. Acetate and propionate also interact with Olfr78/OR51E2 receptors in the juxtaglomerular complex of the kidneys, but with lower potency, to create a negative feedback loop by increasing the renin and angiotensin II levels, which in turn elevates the blood pressure.

## Figures and Tables

**Table 1:** Human trials investigating salt-sensitive hypertension and the microbiome.

Study	Design	N	Population (*n*) **	Treatment Category	Intervention, Duration	HTN Results with Intervention	SCFA Results Associated with HTN	Gut Microbiome Results Associated with HTN
Tilves C et al. [14]	RCT (phase 3) secondary analysis	111	Overweight/obese cancer survivors	NA	NA	NA	Lower fecal butyrate	NA
Li et al. [9]	Prospective cohort	196	Healthy volunteers, pHTN, untreated HTN	NA	NA	NA	NA	Decreased *Bacteroides*/increased *Prevotella*
Ferguson et al. [21]	Prospective cohort	135	Healthy volunteers	NA	NA	Higher BP with HSD		Increased *Prevotella*
Yang et al. [22]	Prospective cohort	17	Healthy volunteers and treated HTN	NA	NA	NA	NA	Reduction in bacterial richness and diversity
Kim et al. [19]	Prospective cohort	40	Healthy volunteers and treated HTN	NA	NA	NA	Lower plasma butyrate	Decreased butyrate-producing bacteria, including *Eubacterium rectale*
Walejko et al. [23]	Prospective cohort	52	Healthy volunteers and HTN	NA	NA	NA	NA	Different gut microbiota taxonomy
Wilck et al. [6]	Non-randomized study (phase 1)	12	Healthy male volunteers	Sodium-restricted diet	HSD, 2 weeks	Increased SBP with HSD	NA	Decreased *Lactobacillus*
Chen et al. [7]	RCT (phase 3) secondary analysis	145	Untreated HTN	Sodium-restricted diet	LSD, 6 weeks	Decreased BP with LSD ***	Lower plasma SCFAs	NA
Jama et al. [20]	RCT (phase 2), crossover	20	Untreated HTN	Prebiotic	HAMSAB * vs. placebo, 3 weeks	Decreased SBP with prebiotic	Decreased plasma acetate and butyrate	Decreased *Parabacteroides* and *Ruminococcus*

* HAMSAB: Prebiotic acetylated and butyrylated high-amylose maize starch supp. ** All studies were in adults >18 years. BP: blood pressure; HTN: hypertension; HSD: high-salt diet; LSD: low-salt diet; NA: not applicable; pHTN: pre-hypertension; RCT: randomized controlled trial; SBP: systolic blood pressure; SCFA: short-chain fatty acid. *** Effect larger in females.

**Table 2:** Animal trials investigating salt-sensitive hypertension and the microbiome *.

Author	Population	Treatment Category	Intervention	HTN Results with Intervention	SCFA Results with Intervention	Microbiome Results with Intervention
* Wilck et al. [6]	Mice, FVB/N	Dietary intervention	HSD	Increased BP	NA	Decreased *Lactobacillus*
Bier et al. [25]	Rats, Dahl salt-sensitive	Dietary intervention	HSD	Increased BP	Increased fecal acetate, propionate, isobutyrate	Unique taxa associated with increased BP ^
Chakraborty et al., 2020 [26]	Rats, Dahl salt-sensitive	Dietary intervention	HSD	Increased BP in a diurnal manner	NA	Microbiota changed in a diurnal manner
Chen et al. [27]	Rats, Sprague-Dawley	Dietary intervention	HSD with fructose	Increased BP	NA	Decreased Firmicutes to Bacteroidetes ratio
* Abais-Battadet et al. [28]	Rats, Dahl salt-sensitive	Dietary intervention	HSD vs. grain diet	Grain diet attenuated HSD-BP increase	NA	NA
* Marques et al. [29]	Mice, C57BI/6 DOCA-salt model	Dietary intervention	High-fiber diet	Decreased BP	NA	Increased acetate-producing Bacteroides
Komatsu et al. [30]	Rats, Dahl salt-sensitive, obese	Dietary intervention	Inulin	Decreased BP	NA	NA
Zhang et al. [31]	Mice, C57BI/6 fed HSD	Dietary intervention	Lactulose	Decreased BP	NA	Increased *Bifidobacterium*
Chakraborty et al., 2018. [32]	Rats, Dahl salt-sensitive fed HSD	Dietary intervention	1,3 butanediol (nutritional supplement)	Decreased BP	NA	Effect independent of microbiota changes
* Wilck et al. [6]	Mice, FVB/N fed HSD	Probiotic	Lactobacillus murinus	Decreased BP	NA	NA
* Marques et al. [29]	Mice, C57BI/6 DOCA-salt model	Postbiotic	Acetate	Decreased BP	NA	Increased acetate-producing Bacteroides acidifaciens
Kim et al. [19]	Mice, C57B16, infused with angiotensin II	Postbiotic	Butyrate	Decreased BP	NA	Increased *Akkermansia muciniphila*
Zhu et al. [33]	Mice, C57BL/6J fed HSD with fructose	Postbiotic	Chlorogenic acid	Decreased BP	NA	Increased *Parabacteroides* and *Klebsiella*
Liu TH et al. [34]	Mice, C57BL/67 fed HSD	Postbiotic	Vitamin K supplementation	Decreased BP	NA	Increased *Dubosiella*
* Abais-Battadet al. [28]	Rats, Dahl salt-sensitive	Live biotherapeutic product	FMT from hypertensive rat to high-grain diet rat	Increased BP	NA	Decreased *Erysipelotrichaceae* and *Parabacteroides gordonii*
Li et al. [9]	Mice, germ-free	Live biotherapeutic product	FMT from hypertensive human patients	Increased BP	NA	NA
Mell et al. [24]	Rats, cases: Dahl salt-sensitive; control: Dahl salt-resistant	Live biotherapeutic product	FMT from salt-resistant rat to salt-sensitive rat fed HSD	Increase BP	Increased plasma acetate	Decreased *Veillonellaceae*
Yan et al. [35]	Rats, Wistar, fed HSD	Live biotherapeutic product	FMT from hypertensive rats	Increased BP	NA	Reduced *Bacteroides* with increased BP

* Studies with multiple interventions. ^ *Pseudomonadales* order, *Christensenellaceae*, *Barnesiellaceae, Eubacteriaceae* families, *Erwinia* and *Anaerofustis* genus, and anaerostipes genera. BP: blood pressure; DOCA: deoxycorticosteroine acetate; FMT: fecal microbiota transplant; HSD: high-salt diet.

## Data Availability

All data generated or analyzed during this study are included in this published article.

## References

[1] Martin S.S., Aday A.W., Almarzooq Z.I., Anderson C.A.M., Arora P., Avery C.L., Baker-Smith C.M., Barone Gibbs B., Beaton A.Z., Boehme A.K. (2024). 2024 Heart Disease and Stroke Statistics: A Report of US and Global Data From the American Heart Association. Circulation.

[2] He F.J., Li J., Macgregor G.A. (2013). Effect of longer term modest salt reduction on blood pressure: Cochrane systematic review and meta-analysis of randomised trials. BMJ.

[3] Bailey M.A., Dhaun N. (2024). Salt Sensitivity: Causes, Consequences, and Recent Advances. Hypertension.

[4] Naqvi S., Asar T.O., Kumar V., Al-Abbasi F.A., Alhayyani S., Kamal M.A., Anwar F. (2021). A cross-talk between gut microbiome, salt and hypertension. Biomed. Pharmacother..

[5] Weinberger M.H., Miller J.Z., Luft F.C., Grim C.E., Fineberg N.S. (1986). Definitions and characteristics of sodium sensitivity and blood pressure resistance. Hypertension.

[6] Wilck N., Matus M.G., Kearney S.M., Olesen S.W., Forslund K., Bartolomaeus H., Haase S., Mähler A., Balogh A., Markó L. (2017). Salt-responsive gut commensal modulates T_H_17 axis and disease. Nature.

[7] Chen L., He F.J., Dong Y., Huang Y., Wang C., Harshfield G.A., Zhu H. (2020). Modest Sodium Reduction Increases Circulating Short-Chain Fatty Acids in Untreated Hypertensives: A Randomized, Double-Blind, Placebo-Controlled Trial. Hypertension.

[8] Avery E.G., Bartolomaeus H., Maifeld A., Marko L., Wiig H., Wilck N., Rosshart S.P., Forslund S.K., Müller D.N. (2021). The Gut Microbiome in Hypertension: Recent Advances and Future Perspectives. Circ. Res..

[9] Li J., Zhao F., Wang Y., Chen J., Tao J., Tian G., Wu S., Liu W., Cui Q., Geng B. (2017). Gut microbiota dysbiosis contributes to the development of hypertension. Microbiome.

[10] Desai M.S., Seekatz A.M., Koropatkin N.M., Kamada N., Hickey C.A., Wolter M., Pudlo N.A., Kitamoto S., Terrapon N., Muller A. (2016). A Dietary Fiber-Deprived Gut Microbiota Degrades the Colonic Mucus Barrier and Enhances Pathogen Susceptibility. Cell.

[11] Jama H.A., Beale A., Shihata W.A., Marques F.Z. (2019). The effect of diet on hypertensive pathology: Is there a link via gut microbiota-driven immunometabolism?. Cardiovasc. Res..

[12] Kaye D.M., Shihata W.A., Jama H.A., Tsyganov K., Ziemann M., Kiriazis H., Horlock D., Vijay A., Giam B., Vinh A. (2020). Deficiency of Prebiotic Fiber and Insufficient Signaling Through Gut Metabolite-Sensing Receptors Leads to Cardiovascular Disease. Circulation.

[13] Xu J., Moore B.N., Pluznick J.L. (2022). Short-Chain Fatty Acid Receptors and Blood Pressure Regulation: Council on Hypertension Mid-Career Award for Research Excellence 2021. Hypertension.

[14] Tilves C., Yeh H.C., Maruthur N., Juraschek S.P., Miller E., White K., Appel L.J., Mueller N.T. (2022). Increases in Circulating and Fecal Butyrate are Associated With Reduced Blood Pressure and Hypertension: Results From the SPIRIT Trial. J. Am. Heart Assoc..

[15] Peters M.D.J., Marnie C., Tricco A.C., Pollock D., Munn Z., Alexander L., McInerney P., Godfrey C.M., Khalil H. (2020). Updated methodological guidance for the conduct of scoping reviews. JBI Evid. Synth..

[16] Cordaillat-Simmons M., Rouanet A., Pot B. (2020). Live biotherapeutic products: The importance of a defined regulatory framework. Exp. Mol. Med..

[17] Davani-Davari D., Negahdaripour M., Karimzadeh I., Seifan M., Mohkam M., Masoumi S.J., Berenjian A., Ghasemi Y. (2019). Prebiotics: Definition, Types, Sources, Mechanisms, and Clinical Applications. Foods.

[18] Al-Habsi N., Al-Khalili M., Haque S.A., Elias M., Olqi N.A., Al Uraimi T. (2024). Health Benefits of Prebiotics, Probiotics, Synbiotics, and Postbiotics. Nutrients.

[19] Kim S., Goel R., Kumar A., Qi Y., Lobaton G., Hosaka K., Mohammed M., Handberg E.M., Richards E.M., Pepine C.J. (2018). Imbalance of gut microbiome and intestinal epithelial barrier dysfunction in patients with high blood pressure. Clin. Sci..

[20] Jama H.A., Rhys-Jones D., Nakai M., Yao C.K., Climie R.E., Sata Y., Anderson D., Creek D.J., Head G.A., Kaye D.M. (2023). Prebiotic intervention with HAMSAB in untreated essential hypertensive patients assessed in a phase II randomized trial. Nat. Cardiovasc. Res..

[21] Ferguson J.F., Aden L.A., Barbaro N.R., Van Beusecum J.P., Xiao L., Simmons A.J., Warden C., Pasic L., Himmel L.E., Washington M.K. (2019). High dietary salt-induced dendritic cell activation underlies microbial dysbiosis-associated hypertension. JCI Insight.

[22] Yang T., Santisteban M.M., Rodriguez V., Li E., Ahmari N., Carvajal J.M., Zadeh M., Gong M., Qi Y., Zubcevic J. (2015). Gut dysbiosis is linked to hypertension. Hypertension.

[23] Walejko J.M., Kim S., Goel R., Handberg E.M., Richards E.M., Pepine C.J., Raizada M.K. (2018). Gut microbiota and serum metabolite differences in African Americans and White Americans with high blood pressure. Int. J. Cardiol..

[24] Mell B., Jala V.R., Mathew A.V., Byun J., Waghulde H., Zhang Y., Haribabu B., Vijay-Kumar M., Pennathur S., Joe B. (2015). Evidence for a link between gut microbiota and hypertension in the Dahl rat. Physiol. Genom..

[25] Bier A., Braun T., Khasbab R., Di Segni A., Grossman E., Haberman Y., Leibowitz A. (2018). A High Salt Diet Modulates the Gut Microbiota and Short Chain Fatty Acids Production in a Salt-Sensitive Hypertension Rat Model. Nutrients.

[26] Chakraborty S., Mandal J., Cheng X., Galla S., Hindupur A., Saha P., Yeoh B.S., Mell B., Yeo J.Y., Vijay-Kumar M. (2020). Diurnal Timing Dependent Alterations in Gut Microbial Composition Are Synchronously Linked to Salt-Sensitive Hypertension and Renal Damage. Hypertension.

[27] Chen Y., Zhu Y., Wu C., Lu A., Deng M., Yu H., Huang C., Wang W., Li C., Zhu Q. (2020). Gut dysbiosis contributes to high fructose-induced salt-sensitive hypertension in Sprague-Dawley rats. Nutrition.

[28] Abais-Battad J.M., Saravia F.L., Lund H., Dasinger J.H., Fehrenbach D.J., Alsheikh A.J., Zemaj J., Kirby J.R., Mattson D.L. (2021). Dietary influences on the Dahl SS rat gut microbiota and its effects on salt-sensitive hypertension and renal damage. Acta Physiol.

[29] Marques F.Z., Nelson E., Chu P.-Y., Horlock D., Fiedler A., Ziemann M., Tan J.K., Kuruppu S., Rajapakse N.W., El-Osta A. (2017). High-Fiber Diet and Acetate Supplementation Change the Gut Microbiota and Prevent the Development of Hypertension and Heart Failure in Hypertensive Mice. Circulation.

[30] Komatsu Y., Aoyama K., Yoneda M., Ashikawa S., Nakano S., Kawai Y., Cui X., Furukawa N., Ikeda K., Nagata K. (2021). The prebiotic fiber inulin ameliorates cardiac, adipose tissue, and hepatic pathology, but exacerbates hypertriglyceridemia in rats with metabolic syndrome. Am. J. Physiol. Heart Circ. Physiol..

[31] Zhang Z., Zhao J., Tian C., Chen X., Li H., Wei X., Lin W., Zheng N., Jiang A., Feng R. (2019). Targeting the Gut Microbiota to Investigate the Mechanism of Lactulose in Negating the Effects of a High-Salt Diet on Hypertension. Mol. Nutr. Food Res..

[32] Chakraborty S., Galla S., Cheng X., Yeo J.Y., Mell B., Singh V., Yeoh B., Saha P., Mathew A.V., Vijay-Kumar M. (2018). Salt-Responsive Metabolite, β-Hydroxybutyrate, Attenuates Hypertension. Cell Rep..

[33] Zhu Q., Zhu Y., Liu Y., Tao Y., Lin Y., Lai S., Liang Z., Chen Y., Chen Y., Wang L. (2022). Moderation of gut microbiota and bile acid metabolism by chlorogenic acid improves high-fructose-induced salt-sensitive hypertension in mice. Food Funct..

[34] Liu T.H., Tao W.C., Liang Q.E., Tu W.Q., Xiao Y., Chen L.G. (2020). Gut Microbiota-Related Evidence Provides New Insights Into the Association Between Activating Transcription Factor 4 and Development of Salt-Induced Hypertension in Mice. Front. Cell Dev. Biol..

[35] Yan X., Jin J., Su X., Yin X., Gao J., Wang X., Zhang S., Bu P., Wang M., Zhang Y. (2020). Intestinal Flora Modulates Blood Pressure by Regulating the Synthesis of Intestinal-Derived Corticosterone in High Salt-Induced Hypertension. Circ. Res..

[36] Natarajan N., Hori D., Flavahan S., Steppan J., Flavahan N.A., Berkowitz D.E., Pluznick J.L. (2016). Microbial short chain fatty acid metabolites lower blood pressure via endothelial G protein-coupled receptor 41. Physiol. Genom..

[37] Pluznick J.L., Protzko R.J., Gevorgyan H., Peterlin Z., Sipos A., Han J., Brunet I., Wan L.X., Rey F., Wang T. (2013). Olfactory receptor responding to gut microbiota-derived signals plays a role in renin secretion and blood pressure regulation. Proc. Natl. Acad. Sci. USA.

[38] Pluznick J.L. (2017). Microbial Short-Chain Fatty Acids and Blood Pressure Regulation. Curr. Hypertens. Rep..

[39] Vinelli V., Biscotti P., Martini D., Del Bo C., Marino M., Meroño T., Nikoloudaki O., Calabrese F.M., Turroni S., Taverniti V. (2022). Effects of Dietary Fibers on Short-Chain Fatty Acids and Gut Microbiota Composition in Healthy Adults: A Systematic Review. Nutrients.

[40] Pepine C.J., Thiel A., Kim S., Handberg E.M., Richards E.M., Dasa O., Mohammed M., Smith S.M., Cooper-DeHoff R.M., Raizada M.K. (2021). Potential of Minocycline for Treatment of Resistant Hypertension. Am. J. Cardiol..

[41] Konopelski P., Konop M., Perlejewski K., Bukowska-Osko I., Radkowski M., Onyszkiewicz M., Jaworska K., Mogilnicka I., Samborowska E., Ufnal M. (2021). Genetically determined hypertensive phenotype affects gut microbiota composition, but not vice versa. J. Hypertens..

